# Supplementary material for: Crosstalk between CXCR4/ACKR3 and EGFR Signaling in Breast Cancer Cells
Source: Int J Mol Sci. 2022 Oct 6;23(19):11887. doi: 10.3390/ijms231911887 (PMC9570020; doi:10.3390/ijms231911887)
Supplement: Supplementary file 1 [file ijms-23-11887-s001.zip › Figures S1-S4.pdf]

Supplemental figures

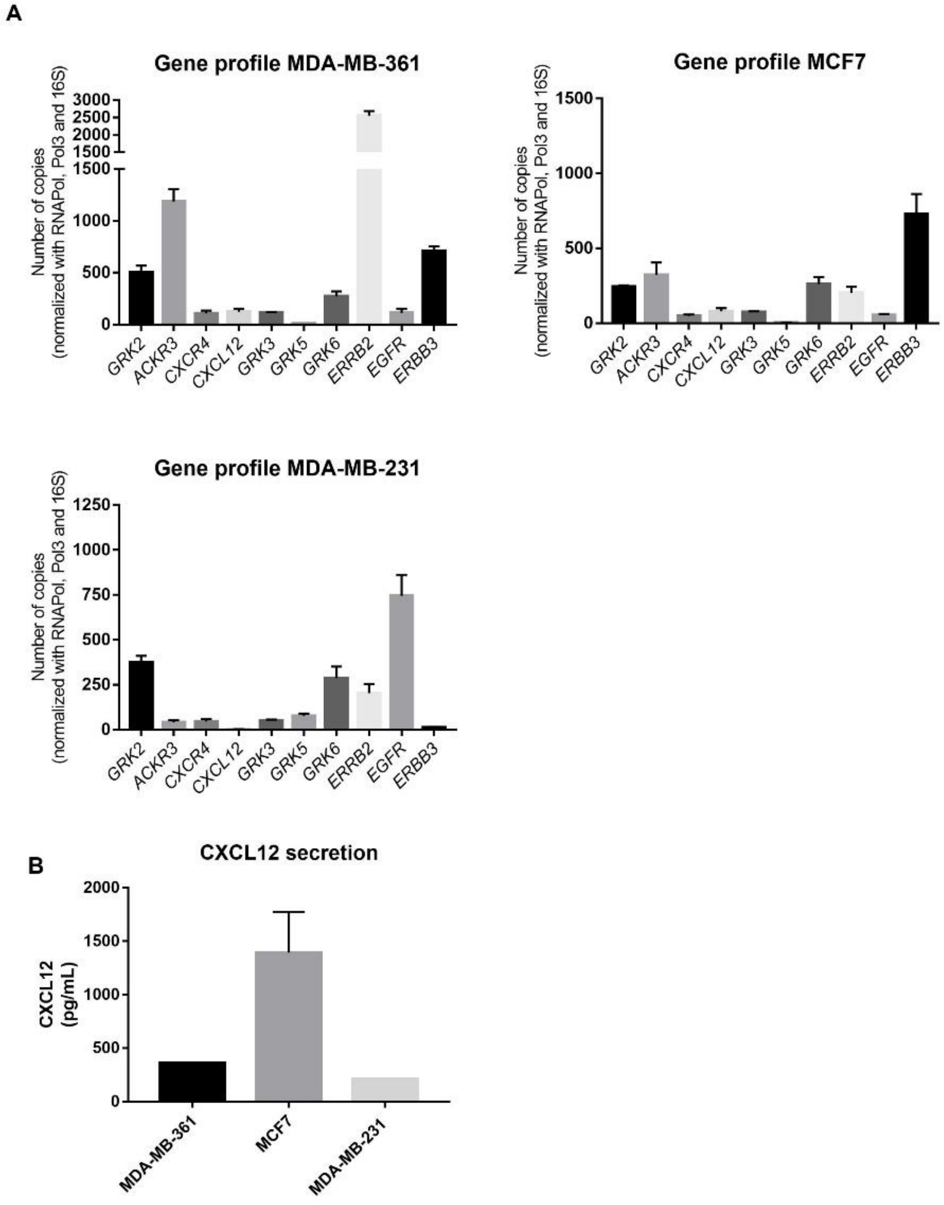

Figure S1. Expression profile of GRK and EGFR (ERBB) family members and of the CXCL12/CXCR4/ACKR3 signaling module in model breast cancer cell lines. **(A)** qRT-PCR analysis of mRNA expression of the indicated genes. mRNA levels were normalized by a geometric mean of RNAPol, Pol3 and 16S. Data are mean±SEM of 4 independent experiments. **(B)**. CXCL12 secretion levels in conditioned media of MCF7, MDA-MB 361 and MDA-MB 231 cell lines determined as described in Materials and Methods. Data are mean ± SEM from 2-3 experiments.

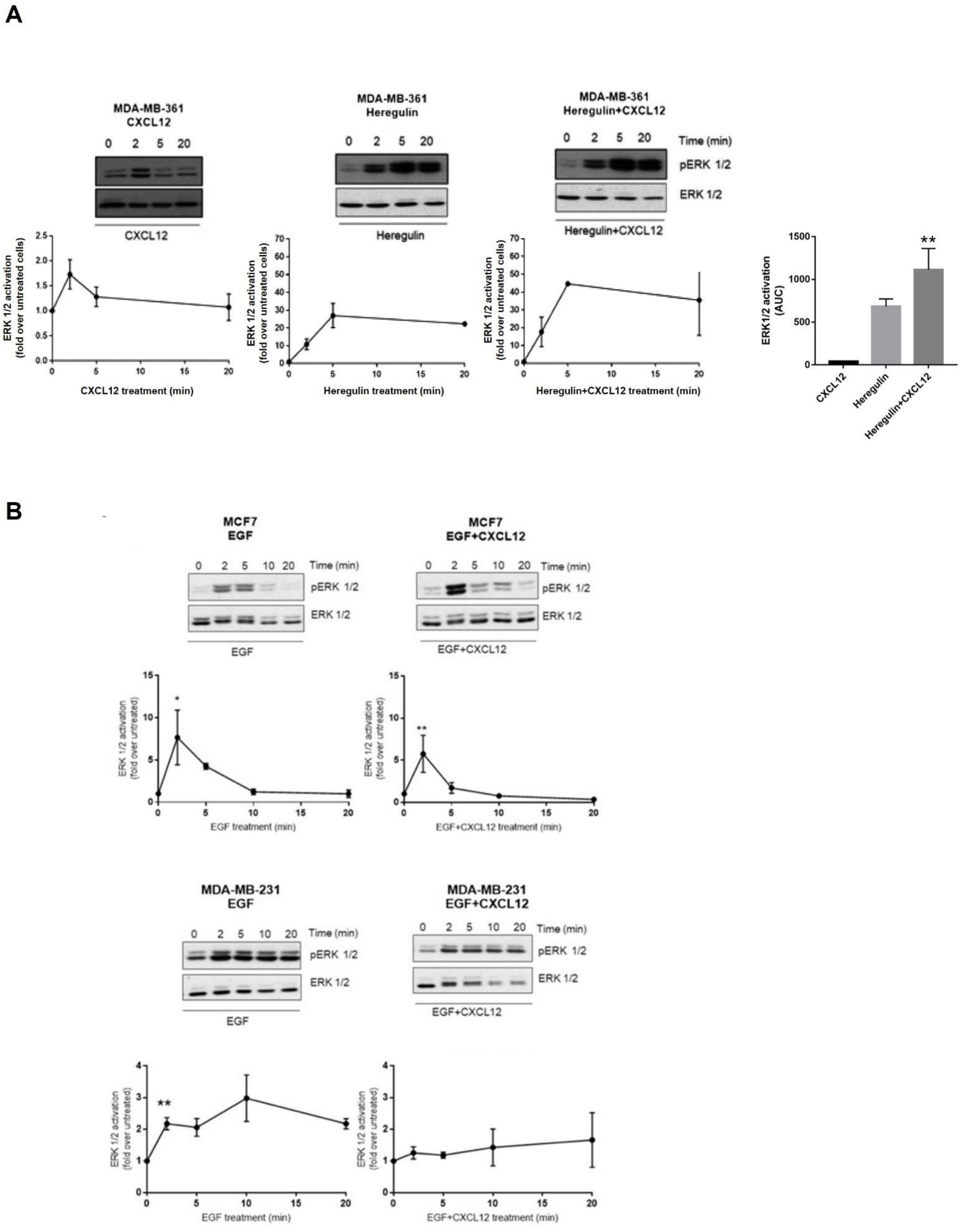
A
MDA-MB-361
CXCL12
0 2 5 20
CXCL12
ERK 1/2 activation
(fold over untreated cells)
CXCL12 treatment (min)
MDA-MB-361
Heregulin
0 2 5 20
Heregulin
ERK 1/2 activation
(fold over untreated cells)
Heregulin treatment (min)
MDA-MB-361
Heregulin+CXCL12
0 2 5 20
Time (min)
pERK 1/2
ERK 1/2
Heregulin+CXCL12
ERK 1/2 activation
(fold over untreated cells)
Heregulin+CXCL12 treatment (min)
ERK1/2 activation
(AUC)
**
CXCL12
Heregulin
Heregulin+CXCL12
B
MCF7
EGF
0 2 5 10 20
Time (min)
pERK 1/2
ERK 1/2
EGF
ERK 1/2 activation
(fold over untreated)
*
EGF treatment (min)
MCF7
EGF+CXCL12
0 2 5 10 20
Time (min)
pERK 1/2
ERK 1/2
EGF+CXCL12
ERK 1/2 activation
(fold over untreated)
**
EGF+CXCL12 treatment (min)
MDA-MB-231
EGF
0 2 5 10 20
Time (min)
pERK 1/2
ERK 1/2
EGF
ERK 1/2 activation
(fold over untreated)
**
EGF treatment (min)
MDA-MB-231
EGF+CXCL12
0 2 5 10 20
Time (min)
pERK 1/2
ERK 1/2
EGF+CXCL12
ERK 1/2 activation
(fold over untreated)
EGF+CXCL12 treatment (min)

Figure S2. (A) Simultaneous activation of the CXCL12/CXCR4/ACKR3 and HER2 pathways fosters the ERK cascade stimulation pattern in MDA-MB-361 cells (ER+ and HER2+). Serum-starved cells were stimulated with 10nM CXCL12, 20ng/mL heregulin or the combination of both ligands for the indicated times. ERK1/2 activation was assessed and plotted over time Data of the area under the curve (AUC) are also represented for comparative statistical analysis. Representative blots are shown. Data are mean±SEM of 3 independent experiments. ** $p < 0.01$, comparing to non-stimulated cells. **(B)** Simultaneous activation of the CXCL12/CXCR4/ACKR3 and EGFR cascades attenuates ERK pathway stimulation pattern in MCF7 (ER+) and MDA-MB-231 (triple negative) breast cancer cells. MCF7 and MDA-MB-231 cells were serum- starved (1%FBS) overnight and stimulated with 10nM CXCL12, 100ng/mL EGF or the combination of both for the indicated times. ERK1/2 activation was assessed in cell lysates by western blot with specific antibodies and data normalized by total ERK levels. No additive but rather antagonistic effects were observed between CXCR4/ACKR3- EGFR pathways for ERK activation. Representative blots are shown. Data are mean±SEM of 2-4 independent experiments. *$p<0.05$ or ** $p< 0.01$, comparing to non-stimulated cells.

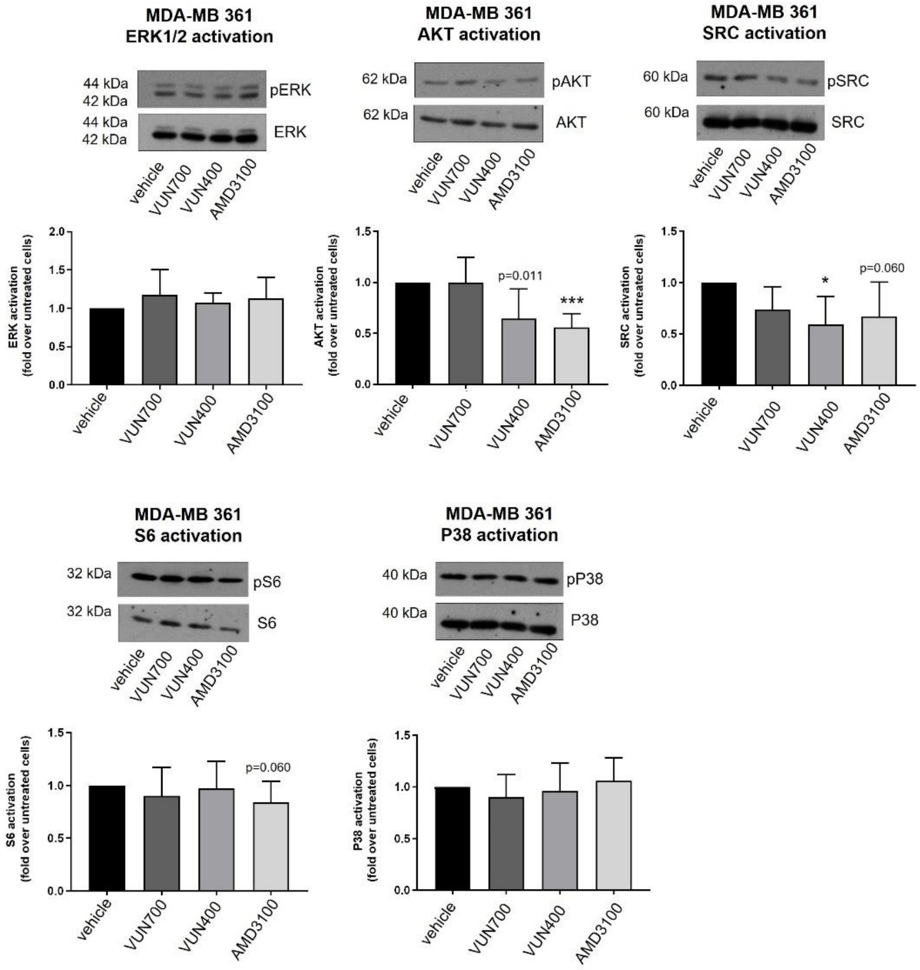

Figure S3. The presence of CXCR4 modulators decreases basal AKT and SRC activation in MDA-MB-361 cells (ER+, Her2+). Cells were treated with the indicated inhibitors for 24h. ERK1/2, AKT, SRC, SRC and p38 activation was assessed in cell lysates by western blot with specific antibodies as detailed in Methods. Representative blots are shown. Data are mean±SEM of 5 independent experiments. * $p < 0.05$ or *** $p < 0.001$, comparing to non-stimulated cells.

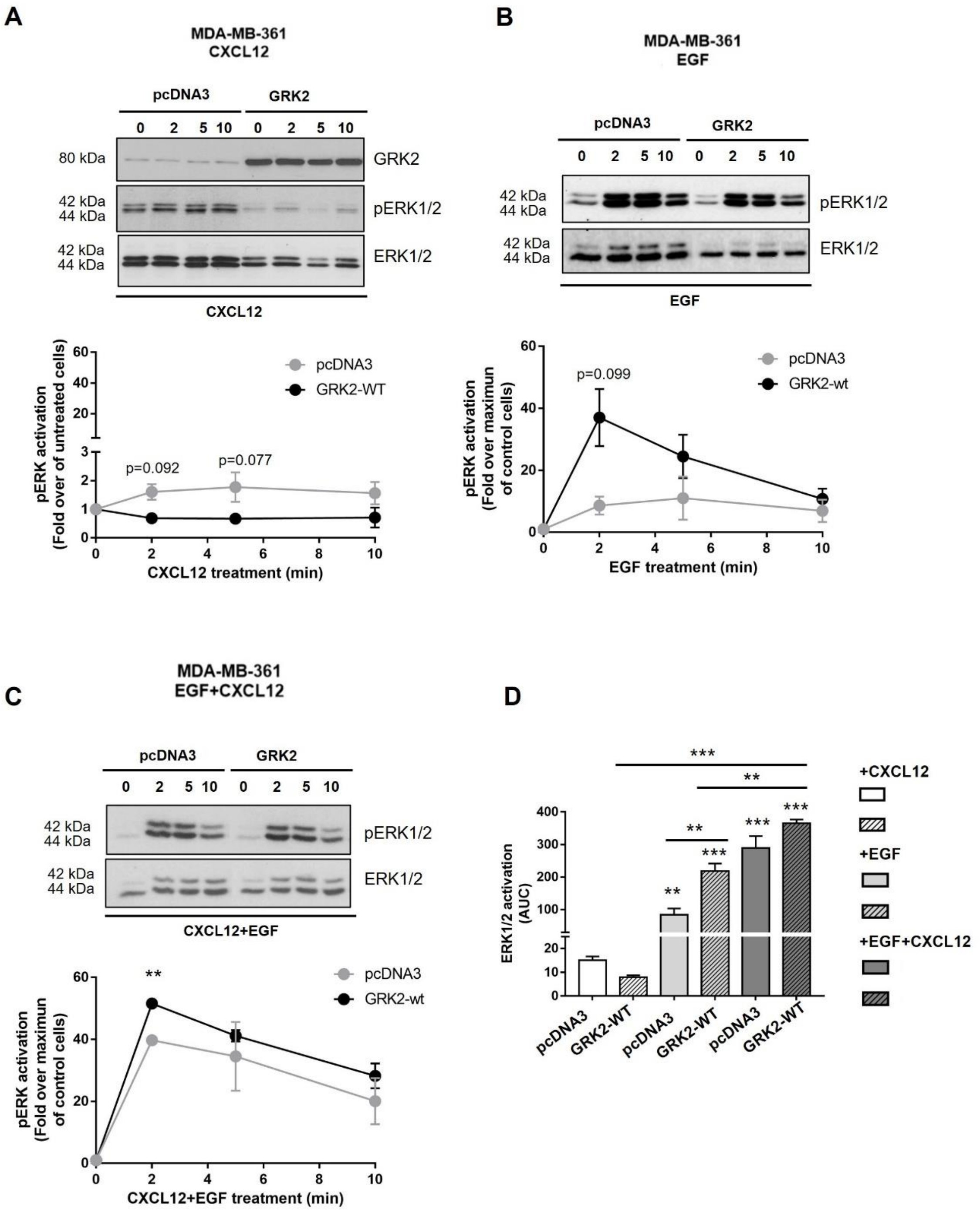

Figure S4. GRK2 overexpression down- regulates ERK1/2 activation by CXCL12 whilst fostering ERK1/2 stimulation by EGF or upon simultaneous activation of the CXCL12/CXCR4/ACKR3 and EGFR pathways in MDA-MB-361 cells (ER+ and HER2+). The GRK2-mediated decrease of ERK1/2 activation In the presence of CXCL12 is relieved upon co-stimulation with EGF, leading to a stronger activation of this pathway. Cells were transiently transfected with the GRK2-WT construct or empty vector. Following serum starvation, cells were stimulated with 10nM CXCL12 (A), 100ng/mL EGF (B) or the combination of both ligands (C) for the indicated times. ERK1/2 activation was assessed and normalized by total ERK protein. GRK2 levels were immunodetected with a specific antibody. The area under the curve (AUC) of each condition is represented (D). Data are mean±SEM of 2-3 independent experiments. ** $p< 0.01$, or *** $p< 0.001$, comparing to non-stimulated cells.
