# Supplementary material for: Crosstalk between CXCR4/ACKR3 and EGFR Signaling in Breast Cancer Cells
Source: Int J Mol Sci. 2022 Oct 6;23(19):11887. doi: 10.3390/ijms231911887 (PMC9570020; doi:10.3390/ijms231911887)
Supplement: Supplementary file 1 [file ijms-23-11887-s001.zip › Tables S1-S4.pdf]

Supplemental Materials and Methods

Table S1. Cell treatment details. For cellular stimulation with ligands, cells were serum-starved overnight and challenged with Heregulin (20 ng/ml), EGF (100 ng/ml) or CXCL12 (10nM) for the indicated times. To determine the influence of cellular components on the signalling dynamics of these ligands, cells were pre-treated with the following inhibitors prior to stimulation.

Treatment	Specifications
AG1478	500nM for 2h
AMD3100	10µM 2h
CMPD101	30µM, 2h
PP2	5µM for 2h
PTX	100µg/mL for 2h
VUN400 (C2)	10µg/mL
VUN700 (2C5)	10µg/mL

Table S2. Sources and experimental conditions of antibodies used

Primary Antibodies

Antigen	Reference	Supplier	Species	Dilution
**AKT**	9272	Cell Signalling	Rabbit	WB (1/1000)
**CXCR4-PE**	306505	Biolegend	Mouse	FC (2.5µl/10^6 cells)
**ACKR3-APC**	331113	Biolegend	Mouse	FC(2.5µl/10^6 cells)
**ERK1**	(C-16): sc-93	Santa Cruz	Rabbit	WB (0.75 mg/ml)
**ERK2**	(c-14): sc-154	Santa Cruz	Rabbit	WB (0.75 mg/ml)
**FAK**	3285	Cell Signalling	Rabbit	WB (1/1000)
**GRK2**	C15 (sc-562)	Santa Cruz	Rabbit	WB (1/1000
**p-AKT (Ser473)**	9271	Cell Signalling	Rabbit	WB (1/1000)
**PARK7/DJ1**	Ab18257	Abcam	Rabbit	WB (1/5000)
**p-Erk1-2 (Thr202/204)**	9101	Cell Signalling	Rabbit	WB (1/1000)
**p-FAK (Tyr397)**	3283	Cell Signalling	Rabbit	WB (1/500)
**p-p38 MAPK (Thr180/Tyr182)**	4631	Cell Signalling	Rabbit	WB (1/500)
**p-S6 ribosomal protein (Ser240/244)**	2215	Cell Signalling	Rabbit	WB (1/1000)
**p-Src Family (Tyr416)**	2101	Cell Signalling	Rabbit	WB (1/1000)
**p38α MAPK**	9218	Cell Signalling	Rabbit	WB (1/1000)
**S6 ribosomal protein (5G10)**	2217	Cell Signalling	Rabbit	WB (1/000)
**Src**	2108	Cell Signalling	Rabbit	WB (1/000)

Secondary antibodies

Name, antigen	Company	Dilution

**Biotin-conjugated donkey anti-mouse IgG**	Jackson Immnunoresearch	WB (1/50000)/ IHC (1/100)
**Biotin-conjugated donkey anti-rabbit IgG**	Jackson Immnunoresearch	WB (1/50000)/ IHC (1/100)
**Soluble rabbit peroxidase-anti-mouse IgG**	Nordic immunology	WB (1/50000)
**Soluble mouse peroxidase-anti-rabbit IgG**	Nordic immunology	WB (1/50000)
**Soluble mouse peroxidase-anti-goat IgG**	Nordic immunology N1	WB (1/50000)
**Streptavidin POD-conjugated**	Roche	WB (1/50000)

Table S3. Primers used for mRNA analysis

Target	Primer	Sequence 5´- 3´
ACKR3	Fw	AAGAAGATGGTACGCCGTGT
	Rv	AGGCAGAGACACGCAGAAG
CXCL12	Fw	GCTGGTCCTCGTGCTGAC
	Rv	GCATGGGCATCTGTAGCTC
CXCR4	Fw	GGTGGTCTATGTTGGCGTCT
	Rv	ACTGACGTTGGCAAAGATGA
EGFR	Fw	TCCCCGTAATTATGTGGTGAC
	Rv	AGGCCCTTCGCACTTCTTAC
ERBB2	Fw	CAGAATGGCTCAGTGACCTG
	Rv	GCAGAAGGGAGGGTCCTTAT
ERBB3	Fw	ACAGCCCCAGATCTGCAC
	Rv	GTTGGGCGAATGTTCTCATC
GRK2	Fw	CATGCACAATCGCTTTGTAGTC
	Rv	GGTCCGAGATTCTCACATGG
GRK3	Fw	AACTTGGCCACGAAGAAGATT
	Rv	CTCAGGCCGTACCTCTGGT
GRK5	Fw	GGTGGGCACTGTTGGCTA
	Rv	CTCAGGCCGTACCTCTGGT
GRK6	Fw	ATCAGCCAGTGCGAAGA
	Rv	CGTGGCACAGAACTCTCG
